# Tarsal Tunnel Mechanosensitivity Is Increased in Patients with Asthma: A Case-Control Study

**DOI:** 10.3390/jcm7120541

**Published:** 2018-12-12

**Authors:** César Calvo-Lobo, Roi Painceira-Villar, Daniel López-López, Vanesa García-Paz, Ricardo Becerro-de-Bengoa-Vallejo, Marta Elena Losa-Iglesias, Patricia Palomo-López

**Affiliations:** 1 Nursing and Physical Therapy Department, Institute of Biomedicine (IBIOMED), Faculty of Health Sciences, Universidad de León, 24401 León, Spain; ccall@unileon.es; 2 Research, Health and Podiatry Unit, Department of Health Sciences. Faculty of Nursing and Podiatry, Universidade da Coruña, 15403 Ferrol, Spain; roi.painceira.villar@udc.es; 3 Departament of Allergology, Complexo Hospitalario Universitario de Ferrol, 15405 Ferrol, Spain; vanesa.garcia.paz@sergas.es; 4 Facultad de Enfermería, Fisioterapia y Podología, Universidad Complutense de Madrid, 28670 Madrid, Spain; ribebeva@ucm.es; 5 Faculty of Health Sciences, Universidad Rey Juan Carlos, 28670 Alcorcón, Spain; marta.losa@urjc.es; 6 Department of Nursing, University Center of Plasencia, University of Extremadura, 10600 Plasencia, Spain; patibiom@unex.es

**Keywords:** asthma, central nervous system sensitization, pain, spirometry, tarsal tunnel syndrome, tibial nerve

## Abstract

**Background:** Based on changes in lung function and musculoskeletal disorders in patients with asthma, this study aimed to compare the tarsal tunnel and fibular bone pressure pain thresholds (PPTs) of patients with asthma and healthy matched-paired controls. **Methods:** A case-control study was performed. One hundred participants were recruited: 50 asthma patients and 50 healthy matched-paired controls. Bilaterally, tarsal tunnel and fibula bone PPTs were registered. **Results:** Statistically significant differences (*p* < 0.01) were shown bilaterally for tarsal tunnel PPT. With the exception of fibula PPT (*p* > 0.05), asthma patients presented less tarsal tunnel PPT than healthy participants. Statistically significant differences (*p* < 0.05) were shown for two linear regression prediction models of the right (*R*^2^ = 0.279) and left (*R*^2^ = 0.249) tarsal tunnels PPTs as dependent variables, and based on sex, group, contralateral tarsal tunnel PPT and ipsilateral fibula PPT as independent variables. **Conclusions:** The study findings showed that a bilateral tarsal tunnel mechanosensitivity increase is exhibited in patients diagnosed with asthma. The presence of asthma may bilaterally predict the PPT of tarsal tunnel. These findings may suggest the presence of central sensitization in asthma patients, which could clinically predispose them to musculoskeletal disorders, such as tarsal tunnel syndrome.

## 1. Introduction

Worldwide, asthma is considered a common chronic inflammatory condition which presents a significant impairment of the airways and lung function [1]. Changes in quality of life [2], depression, and/or anxiety [3], and behavioral, psychological, and social impairments [4] have been reported in these patients. Indeed, physical, and psychological symptoms associated with asthma should be deeply investigated in order to increase scientific knowledge about their influence on asthma status and the well-being of asthma patients [2].

Lung function may be assessed by means of spirometry [5]. The most frequent used spirometry parameters in patients diagnosed with asthma may be the forced expiratory volume during 1 s (FEV_1_) and the forced vital capacity (FVC), as well as the FEV_1_/FVC coefficient due to reflect airway alterations and the prediction of physiological measurements [6]. 

Patients who suffer from asthma frequently present postural alterations and musculoskeletal disorders [7,8]. Forwarded head and/or shoulders posture, decreased chest wall mobility, reduced shoulder internal rotation, and decreased dorsal spine flexibility with respect to healthy subjects were shown as the most frequent posture impairments [7]. Regarding musculoskeletal disorders in asthma patients, there have been reports of temporomandibular joint and neck pain and palpatory tenderness of some upper quadrant muscles, such as the sternocleidomastoid, paravertebral muscles, medial and lateral pterygoids, and trapezius muscle [8]. Thus, a possible central sensitization process is assumed to be a key focus to explain the mechanisms which produce the tenderness, pain, and musculoskeletal disorders in asthma patients [7,8,9].

Indeed, this central sensitization may originate from the airways autonomic innervation by means of the parasympathetic nervous system, which may be altered due to pharmacological interventions and physiological changes in patients with asthma [10,11]. Synapses of the central nervous system, interaction with vagal afferent nerves subtypes implicated in the regulation of airways function, and the integration of their convergence at key locations of the brain may lead to a central sensitization process of the somatic pathways which increases pain sensation and the pressure pain threshold (PPT) [9]. In addition, increased tenderness and pain seemed to be common complaints associated with postural and musculoskeletal conditions among asthma patients [7,8].

Regarding this central sensitization process in asthma patients, tenderness, pain, and musculoskeletal disorders have previously been evaluated in the body’s upper quadrant [7,8,9], but there is a lack of knowledge about the extent of PPT alterations in the lower limbs of asthma patients. Recently, ankle alterations were related to reduced contractility of the diaphragm muscle [12], which may be also be a possible mechanism of foot sensitization in asthma patients. PPTs of the foot bones and soft tissues have shown the presence of central sensitization in patients with other medical conditions such as migraine [13], low back pain [14], knee osteoarthritis [15], and irritable bowel syndrome [16]. Considering the bone and soft tissue structures of the foot, the lateral malleolus for assessing bone PPT [17] and the tarsal tunnel (including plantar flexor tendons, blood vessels, and tibial nerve trunk) for evaluation of soft tissue PPT [18,19,20,21,22,23] may be considered as one of the most useful sites to assess PPT by means of algometry. 

We therefore hypothesized that the presence of central sensitization in asthma patients might exhibit a higher bone and soft tissue mechanosensitivity at the lateral malleolus and tarsal tunnel, thus producing a lower PPT than among healthy individuals. The main aim of this research was to determine how PPT of the lateral malleolus and tarsal tunnel differed between asthma patients and healthy matched-paired controls. 

## 2. Materials and Methods

### 2.1. Study Design

A case-control study design was used to compare the lateral fibular malleolus and tarsal tunnel soft tissue PPTs of patients diagnosed with asthma and healthy matched-paired controls. In accordance with this design and the recommended guidelines for reporting observational studies, The Strengthening the Reporting of Observational Studies in Epidemiology (STROBE) criteria and checklist were applied [24].

### 2.2. Ethical Statement

The Research Ethics Committee of Universidade da Coruña (Spain) provided a positive assessment for this study. Prior to the start of the study, all subjects signed informed consent forms. In addition, all ethical regulations for human experimentation, as well as the Helsinki Declaration provisions were complied with [25,26].

### 2.3. Sample Size Calculation

Differences between two groups for independent samples were used to calculate the sample size by means of the G*Power software (3.1.9.2v; Statistical Power Analyses for Windows, Universität Düsseldorf, German) according to the ankle tarsal tunnel PPT (kg/cm^2^) of a pilot study (*n* = 40) with two groups (mean ± SD), 20 patients diagnosed with asthma (case group; 3.98 ± 1.41 kg/cm^2^) and 20 healthy matched-paired subjects (control group, 4.60 ± 1.00 kg/cm^2^). Furthermore, statistical parameters, such as one-tailed hypothesis according to a lower PPT secondary to the central sensitization process in asthmatic patients with respect to healthy subjects [7,8,9], effect size = 0.50, α-error = 0.05, power (1-β error probability) = 0.80, and allocation ratio (N2/N1) = 1, were used for the sample size calculation. Thus, the total sample size consisted of 98 subjects, of whom 49 patients had been diagnosed with asthma and 49 were healthy matched-paired participants.

### 2.4. Sample

A consecutive sampling method was applied in order to recruit the participants from the Ferrol University Hospital of Universidade da Coruña and from a private clinic (A Coruña, Spain). Inclusion criteria were people between 18 and 65 years of age that agreed to sign the informed consent form, that were non-smokers, and that had not undergone anti-allergic immunotherapy interventions. The case group comprised patients diagnosed with asthma or allergic asthma by an experienced allergist physician. They presented the typical clinical symptomatology of asthma diagnosis and had undergone a positive lung function bronchodilator exam showing an FEV_1_ parameter greater than 200 mL and 12% with regard to baseline values [5,6]. In addition, this group included individuals that had been prescribed bronchodilators interventions by allergists. The control group comprised healthy matched-paired subjects. Excluded were those under age 18 years or more than 65 years of age; those who refused to sign the informed consent form; active smokers; and individuals being treated with allergy immunotherapy, with reduced ambulation capacity, or with systemic conditions, neuropathies, musculoskeletal conditions (i.e., sprains, tendinopathies, muscle injuries or pain disorders), fractures, diagnosed psychiatric illnesses, or malignant tumors [27]. 

### 2.5. Socio-Demographic and Descriptive Data

Quantitative socio-demographic and descriptive data were recorded, including age (y), height (m), weight (kg), and body mass index (BMI according to the Quetelet index and measured as kg/m^2^) [28]. In addition, physical activity scores were self-reported by the participants using the Spanish validated International Physical Activity Questionnaire (IPAQ) to calculate the total index of metabolic equivalents per minute/week (METS/min/week) during four levels of physical activity. The reliability of this questionnaire was shown to be excellent with an intra-class correlation coefficient (ICC) of 0.93 [29].

Categorical data were collected, including civil status (i.e., single/divorced/widowed/couple/married), professional activity (student/freelancer/employed/unemployed/retired), plantar orthosis (dichotomous variable with yes or no categories), sex (dichotomous variable with male or female categories), as well as physical activity levels evaluated by means of the Spanish IPAQ categories (according to “low” level < 600 METS, “moderate” level between 600 and 3000 METS, and “vigorous” level > 3000 METS) [29].

### 2.6. Primary Outcome Measurements

PPT was evaluated by an analogic manual mechanical algometer (FDK/FDN series, Wagner Instrument, Greenwich, CT, England) with a range between 0 and 10 kg/cm^2^. This was shown to be an excellent, reliable and valid tool for measuring mechanosensitivity, showing a variation coefficient of 10.3%, an ICC of 0.91, a standard error for measurement of 0.19 kg/cm^2^, and a minimal detectable change (MDC) of 0.54 kg/cm^2^ [30]. This tool consisted of a circular platform with an area of 1 cm^2^ area, which was perpendicularly applied to generate progressive pressure on the skin at a 1 kg/sec rate. Subjects were required to indicate the PPT when the mechanosensitivity stimulus felt uncomfortable or painful. Neutral ankle dorsiflexion and supine decubitus position were applied. The mean of three repeated measurements, at 30–60 s intervals, was used for the analysis data based on earlier studies [17,18,19,20,21,22,23].

In fact, tarsal tunnel PPT was assessed in order to evaluate plantar flexor tendons, blood vessels, and tibial nerve trunk soft tissue mechanosensitivity located at 1 cm posterior to the tibial bone medial styloid process [18,19,20,21,22,23]. Fibula PPT was assessed in order to evaluate bone mechanosensitivity located at the center of the ankle lateral malleolus [17].

### 2.7. Secondary Outcome Measurements

Spirometry parameters were assessed by an experienced and specialist allergist to determine the airways flow restriction used the Datospir-600 Touch tool (software from SIBELMED e-20; Rosselló, 08026 Barcelona, Spain) [5]. FVC, FEV_1_, and FEV_1_/FVC parameters were measured as percentages (%) since these values are considered among the most useful spirometer values for physiological measurements prediction in patients diagnosed with asthma [6]. These values reflected lung function and were previously correlated with an *r* of 0.747 for chest wall expansion [31]. These spirometry values showed a good reliability with an ICC from 0.786 to 0.929 [32].

### 2.8. Statistics

Statistical procedures were performed using the Statistical Package for Social sciences (SPSS 24.0 version; IBM Corp., Armonk, NY, USA) considering a 0.05 α error (*p*-value < 0.05 as statistically significant) for a 95% confidence interval (CI). 

Regarding quantitative data, the Kolmogorov-Smirnov test (using the significance of the Lilliefors correction) was used to assess normality distribution. Parametric data (according to a Kolmogorov-Smirnov test with a *p*-value ≥ 0.05) were detailed as mean ± standard deviation (SD) in conjunction with range (minimum–maximum), as well as differences between case and control groups were determined by the independent samples Student’s *t* test (according to the Levene’s test for evaluating equality of variance). Non-parametric data (according to a Kolmogorov-Smirnov test with a *p*-value < 0.05) were detailed like median ± interquartile range (IR) in conjunction with range (minimum–maximum). Differences between case and control groups were determined by the independent samples Mann-Whitney *U* test. In addition, box-plots were added in order to illustrate bilaterally tibial bone and tarsal tunnel PPT differences between case and control groups.

Considering categorical data, frequencies in conjunction with percentages were used to detail these values. Differences between case and control groups were determined by the chi-square (χ^2^) test, except for the dichotomous variables (sex and plantar orthosis), which were analyzed by the Fisher’s exact test. 

In addition, a multivariate predictive analysis was carried out by means of linear regression in order to predict the statistically significant differences between the two groups according to the prior described analyses (bilateral tarsal tunnel PPT). Linear regression analysis was performed applying the stepwise selection method and the *R*^2^ coefficient to determine the quality adjustment. Descriptive data including quantitative (age, sex, weight, height, BMI, and IPAQ scores) and categorical data (professional activity, civil status, sex, plantar orthosis, and IPAQ category), as well as outcome measurements (such as bilateral fibula PPTs, FVC, FEV_1_, and FEV_1_/FVC) were included as independent variables. Bilaterally, tarsal tunnel PPTs were considered as the dependent variables.

## 3. Results

### 3.1. Socio-Demographic and Descriptive Data

One hundred participants were recruited: Half were patients diagnosed with asthma (case group; *n* = 50) and half were matched-paired healthy subjects (control group; *n* = 50) with an age distribution from 19 to 65 years old. The sample included 36 (36%) males and 64 (64%) females. There were no statistical significant differences (*p* > 0.05) between the case and control groups for all quantitative (Table 1) and categorical (Table 2) socio-demographic and descriptive data. 

### 3.2. Primary Outcome Measurements

**Figure 1:**
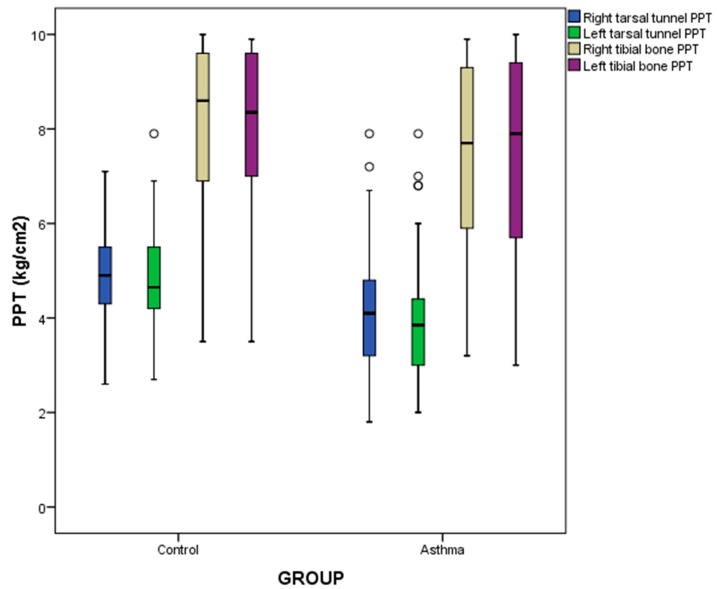
Box-plots to illustrate bilaterally tibial bone and tarsal tunnel PPT differences between patients diagnosed with asthma and healthy matched-paired controls. Abbreviations: PPT, pressure pain threshold.

Regarding PPT outcome measurements (Table 3 and Figure 1), statistically significant differences (*p* < 0.01) were found bilaterally for tarsal tunnel PPT, showing that patients with asthma presented a decreased tarsal tunnel PPT when compared to healthy participants, but not for fibula PPT (*p* > 0.05). 

### 3.3. Secondary Outcome Measurements

Considering spirometry parameters (Table 3), FEV_1_/FVC showed statistically significant differences (*p* = 0.003), demonstrating lower values for patients with asthma with respect to healthy controls, but not for FEV_1_ and FVC (*p* > 0.05), separately. 

### 3.4. Multivariate Predictive Analysis of Tarsal Tunnel PPT

A multivariate regression analysis was carried out for bilateral tarsal tunnel PPT (Table 4) because this measurement was the primary outcome and the only statistically significant difference between case and control groups (Table 3 and Figure 1). The model of linear regression for predicting tarsal tunnel PPT showed statistically significant differences (*p* < 0.05) for two prediction models of the right (*R*^2^ = 0.279) and left (*R*^2^ = 0.249) tarsal tunnels PPTs based on sex, group, contralateral tarsal tunnel PPT, and ipsilateral fibular bone PPT independent variables. The rest of independent variables were excluded from the prediction models. Therefore, bilateral tarsal tunnel PPT measurements in our sample were not predicted or influenced by the rest of independent variables whether they were quantitative (age, weight, height, BMI and IPAQ scores) or categorical (professional activity, civil status, plantar orthosis, and IPAQ category) sociodemographic and descriptive data, as well as lung function parameters (FVC, FEV_1_, and FEV_1_/FVC).

## 4. Discussion

According to the authors’ knowledge, this research study may be the first study to find that asthma patients experienced greater bilateral mechanosensitivity of the tarsal tunnel than healthy matched-paired subjects. This relationship may be a main focus for understanding central pain mechanisms and designing future strategies for central sensitization prevention in asthma patients [7,8,9].

### 4.1. Tarsal Tunnel Mechanosensitivity in Asthma Patients

Despite prior studies suggesting the possible presence of greater tenderness, pain and musculoskeletal disorders of the upper-body quarter among asthma patients [7,8], our study findings showed the possible influence of the central sensitization mechanisms in the lower limbs on lower PPT of the tarsal tunnel in asthma patients when compared to healthy participants. The bilateral tarsal tunnel PPT differences (varied from 0.69 kg/cm^2^ to 0.80 kg/cm^2^ according to the results of Table 3) between asthma and healthy subjects were higher than the MDC (0.54 kg/cm^2^) proposed by Koo et al. [30]. Thus, our findings suggest a strengthened association of higher mechanosensitivity of soft tissues in asthmatic patients with prediction models influenced by the presence of asthma, but not for the bone tissue of the fibular malleolus. We hypothesized that a plausible explanation in order to determine soft tissue by non-bone central sensitization may be that the tarsal tunnel includes flexor hallucis longus, flexor digitorum longus, and posterior tibialis tendons, as well as blood vessels (tibialis posterior venoms and arteries) and nerve trunks (tibial nerve) [33]. These soft tissues may present a greater sensitization than the fibular bone tissue due to the airways autonomic innervation seemed to be derived originally from the parasympathetic nervous system in conjunction with the vagal afferents that regulated airway muscle tone, glandular secretion, and blood-vessel changes, as well as may provide nociceptive inputs to the central nervous system secondary to asthma physiological alterations and pharmacologic interventions [9].

Greater tenderness seemed to be associated with pain and musculoskeletal conditions in asthma patients [7,8], which may be related to a poorer response to interventions in order to reduce central sensitization [34] and a higher quality of life impairment secondary to pain disorders in asthmatic patients [2]. Thus, our findings presented an important clinical relevance for the overall well-being of asthma patients due to the presence of greater tarsal tunnel mechanosensitivity. This mechanosensitivity may suggest the presence of central sensitization, which could clinically predispose asthma patients to musculoskeletal disorders, such as tarsal tunnel syndrome, plantar heel pain, or medial tibial pain [20,21,22,23]. 

In fact, prior case reports in patients who suffered from asthma have described unexplained foot pain and numbness [35], as well as lower limb weakness and pain [36]. Nevertheless, to the authors’ knowledge, the prevalence of foot pain in people with asthma seems to be underreported. Future studies addressing this prevalence should be carried out so that physicians can know when an evaluation of foot pain should be included in clinical consultations with asthma patients.

According to the inclusion criteria, individuals undergoing bronchodilator treatment as prescribed by an allergist were included in the case group. This pharmacological treatment may alter central sensitization mechanisms of asthma patients by autonomic and central nervous system pathways [10,11].

### 4.2. Lung Function

As for the spirometry parameters, the FEV_1_/FVC parameter showed lower values in asthma patients than in healthy subjects based on physiological alterations and airways impairment reported in previous research studies of asthma patients [5,6]. Although patients diagnosed with asthma or allergic asthma were included based on an experienced allergists’ diagnosis, considering the typical clinical symptomatology of asthma diagnosis, as well as a positive lung function bronchodilator test with the FEV_1_ parameter higher than 200 mL and 12% values with respect to baseline measurements [5,6]. The case group included individuals who had been prescribed bronchodilators interventions by their allergists. The spirometry values in our sample were not clinically relevant and were close to baseline values during bronchodilator tests at the moment of PPT evaluation. That finding may be due to the effects of bronchodilator interventions in asthma patients [10,11].

### 4.3. Clinical Implications and Future Studies

In the future, randomized controlled clinical trials of potential pharmacologic treatments, such as GABAergic [37], opioid [38], and serotonergic [39] interventions, as well as tachykinin [40] and glutamate [41,42] receptor blockade treatments should be considered in order to control central sensitization in asthma patients [9]. In addition to a PPT evaluation, pharmacologic interventions for central sensitization should be assessed by quantitative sensory testing, provoked hyperalgesia or allodynia, pain temporal summation, pain spatial summation, descending pain modulation, pain expansion, offset analgesia, and referred pain patterns [43]. Additionally, conservative treatments for patients with musculoskeletal disorders associated with asthma should include education in pain neuroscience, exercise therapy, and cognitive behavioral therapy. All are promising interventions for decreased central sensitization [34].

Based on prior case-control studies’ methodology for evaluating mechanosensitivity [44], our findings showed PPT differences between asthma and healthy subjects. These findings may be used as the minimum clinical relevant values for mechanosensitivity evaluation of the right (PPT = 0.69 kg/cm^2^) and left (PPT = 0.80 kg/cm^2^) tarsal tunnels after interventions in asthma patients because these differences were greater than the recommended MDC values [30].

### 4.4. Limitations

Some possible limitations should be taken into account in this study. First, the consecutive sampling method may be a possible limitation and randomization procedures should be applied to recruit samples for future studies. Second, the age distribution for our study participants only ranged from 19 to 65 years. Age distribution including older adults should be considered for future studies due to the high prevalence among older adults of musculoskeletal disorders associated with central sensitization [45]. 

## 5. Conclusions

The study findings showed that bilateral tarsal tunnel mechanosensitivity increased in asthma patients diagnosed. The presence of asthma may bilaterally predict the PPT of the tarsal tunnel.

## Figures and Tables

**Table 1:** Quantitative sociodemographic and descriptive data for patients diagnosed with asthma, healthy matched-paired controls and total sample.

Quantitative Data	Total Group	Asthma	Control	*p*-Value
	(*n* = 100)	(*n* = 50)	(*n* = 50)	
Age (years)	39.35 ± 12.25	37.22 ± 11.94	41.48 ± 12.31	0.082 *
	(19–65)	(20–65)	(19–65)	
Weight (kg)	70.64 ± 14.33	70.90 ± 15.84	70.38 ± 12.80	0.857 *
	(47–120)	(48–120)	(47–96)	
Height (m)	1.64 ± 0.15	1.66 ± 0.15	1.64 ± 0.13	0.305 ^†^
	(1.50–1.97)	(1.53–1.97)	(1.50–1.87)	
BMI (kg/m^2^)	24.81 ± 5.61	24.43 ± 6.03	24.81 ± 7.00	0.398 ^†^
	(17.30–39.18)	(18.41–39.18)	(17.30–34.72)	
IPAQ (METS/min/week)	2119.50 ± 3620.25	1524.00 ± 3391.13	2772.00 ± 3365.25	0.128 ^†^
	(0–15,918)	(0–15,918)	(0–15,243)	

Abbreviations: BMI, body mass index; IPAQ, International Physical Activity Questionnaire; METs, metabolic equivalent index per week; * Mean ± standard deviation, range (min–max) and Student’s *t*-test for independent samples were applied according to a parametric distribution (Kolmogorov-Smirnov test with a *p*-value ≥ 0.05); ^†^ Median ± interquartile range, range (min–max) and Mann-Whitney *U* test were used according to a non-parametric distribution (Kolmogorov-Smirnov test with a *p*-value < 0.05); In all the analyses, *p* < 0.05 (with a 95% confidence interval) was considered statistically significant.

**Table 2:** Categorical sociodemographic and descriptive data for patients diagnosed with asthma, healthy matched-paired controls and total sample.

Categorical Data		Total Group	Asthma	Control	*p*-Value
		(*n* = 100)	(*n* = 50)	(*n* = 50)	
Professional activity	student	15 (15%)	8 (16%)	7 (14%)	0.440 ^‡^
	freeland	12 (12%)	7 (14%)	5 (10%)	
	employed	58 (58%)	28 (56%)	30 (60%)	
	unemployed	8 (8%)	2 (4%)	6 (12%)	
	retired	7 (7%)	5 (10%)	2 (4%)	
Civil status	single	27 (27%)	12 (24%)	15 (30%)	0.894 ^‡^
	divorced	4 (4%)	2 (4%)	2 (4%)	
	widowed	0 (0%)	0 (0%)	0 (0%)	
	couple	16 (16%)	9 (18%)	7 (14%)	
	married	53 (53%)	27 (54%)	26 (52%)	
IPAQ category *	low	25 (25%)	16 (32%)	9 (18%)	0.264 ^‡^
	moderate	43 (43%)	19 (38%)	24 (48%)	
	vigorous	32 (32%)	15 (30%)	17 (34%)	
Sex	Male	36 (36%)	18 (36%)	18 (36%)	1.000 ^†^
	Female	64 (64%)	32 (64%)	32 (64%)	
Plantar orthosis	Yes	12 (12%)	7 (14%)	5 (10%)	0.760 ^†^
	No	88 (88%)	43 (86%)	45 (90%)	

Abbreviations: METs, metabolic equivalent index per week; IPAQ, International Physical Activity Questionnaire; ^‡^ Frequency, percentage (%) and chi-squared test (χ^2^) were utilized; ^†^ Frequency, percentage (%) and Fisher exact test (χ^2^) were utilized; * Physical activity levels were divided into “low” with less than 600 METS, “moderate” from 600 to 3000 METS, and “vigorous” with more than 3000 METS according to the IPAQ. METS were calculated as the total index of metabolic equivalents per minute/week during four different levels of physical activity [29]; In all the analyses, *p* < 0.05 (with a 95% confidence interval) was considered statistically significant.

**Table 3:** Comparisons of outcome measurements between patients diagnosed with asthma and healthy matched-paired controls.

Outcome Measurements	Total Group	Asthma	Control	*p*-Value Asthma vs. Control
	(*n* = 100)	(*n* = 50)	(*n* = 50)	
Right tarsal tunnel PPT (kg/cm^2^)	4.58 ± 1.21	4.24 ± 1.32	4.93 ± 0.99	**0.004** *
	(1.80–7.90)	(1.80–7.90)	(2.60–7.10)	
Left tarsal tunnel PPT (kg/cm^2^)	4.20 ± 1.50	3.85 ± 1.45	4.65 ± 1.33	**<0.001** ^†^
	(2.00–7.90)	(2.00–7.90)	(2.70–7.90)	
Right fibular bone PPT (kg/cm^2^)	8.20 ± 3.12	7.70 ± 3.43	8.60 ± 2.72	0.097 ^†^
	(3.20–10.00)	(3.20–9.90)	(3.50–10.00)	
Left fibular bone PPT (kg/cm^2^)	8.20 ± 3.07	7.90 ± 3.73	8.35 ± 2.63	0.102 ^†^
	(3.00–10.00)	(3.00–10.00)	(3.50–9.90)	
FVC (%)	96.00 ± 13.00	95.50 ± 15.75	96.50 ± 12.25	0.907 ^†^
	(64–170)	(64–113)	(80–170)	
FEV_1_ (%)	100.13 ± 11.16	98.48 ± 12.21	101.78 ± 9.84	0.140 *
	(61–141)	(61–121)	(84–141)	
FEV_1_/FVC (%)	103.00 ± 10.75	100.00 ± 11.00	105.50 ± 10.00	**0.003** ^†^
	(85–123)	(85–122)	(94–123)	

Abbreviations: FEV_1_, forced expiratory volume in one second; FVC, forced vital capacity; PPT, pressure pain threshold; * Mean ± standard deviation, range (min–max) and Student’s *t*-test for independent samples were used; ^†^ Median ± interquartile range, range (min–max) and Mann-Whitney *U* test were used; In all the analyses, *p* < 0.05 (with a 95% confidence interval) was considered statistically significant (bold).

**Table 4:** Multivariate predictive analysis of bilateral tarsal tunnels PPTs in patients with asthma and healthy matched-paired controls.

Parameter	Model	*R*^2^ Change	Model *R*^2^
Right tarsal tunnel PPT (kg/cm^2^)	5.594		0.279
	−0.809 * Sex	0.145 ^‡^	
	−0.613 * Group	0.083 ^‡^	
	+0.158 * Left tarsal tunnel PPT	0.051 ^†^	
Left tarsal tunnel PPT (kg/cm^2^)	3.748		
	+0.266 * Left fibular bone PPT	0.189 ^‡^	
	−0.617 * Group	0.060 ^‡^	0.249

Abbreviations: PPT, pressure pain threshold; * Multiplay: Group (control = 1; asthma = 2); Left tarsal tunnel PPT (kg/cm^2^); Left tibial bone PPT (kg/cm^2^); Sex (male = 1; female = 2); ^†^ *p*-value < 0.05 for a 95% confidence interval was shown; ^‡^ *p*-value < 0.01 for a 95% confidence interval was shown.
